# Efficient workflow for the investigation of the catalytic cycle of water oxidation catalysts: Combining GFN‐xTB and density functional theory

**DOI:** 10.1002/jcc.26721

**Published:** 2021-07-18

**Authors:** Jan Paul Menzel, Martijn Kloppenburg, Jelena Belić, Huub J. M. de Groot, Lucas Visscher, Francesco Buda

**Affiliations:** ^1^ Leiden Institute of Chemistry Leiden University Leiden The Netherlands; ^2^ Department of Chemistry and Pharmaceutical Sciences Vrije Universiteit Amsterdam Amsterdam The Netherlands

**Keywords:** density functional theory, free energy calculation, GFN‐xTB, transition metal complex, water oxidation

## Abstract

Photocatalytic water oxidation remains the bottleneck in many artificial photosynthesis devices. The efficiency of this challenging process is inherently linked to the thermodynamic and electronic properties of the chromophore and the water oxidation catalyst (WOC). Computational investigations can facilitate the search for favorable chromophore‐catalyst combinations. However, this remains a demanding task due to the requirements on the computational method that should be able to correctly describe different spin and oxidation states of the transition metal, the influence of solvation and the different rates of the charge transfer and water oxidation processes. To determine a suitable method with favorable cost/accuracy ratios, the full catalytic cycle of a molecular ruthenium based WOC is investigated using different computational methods, including density functional theory (DFT) with different functionals (GGA, Hybrid, Double Hybrid) as well as the semi‐empirical tight binding approach GFN‐xTB. A workflow with low computational cost is proposed that combines GFN‐xTB and DFT and provides reliable results. GFN‐xTB geometries and frequencies combined with single‐point DFT energies give free energy changes along the catalytic cycle that closely follow the full DFT results and show satisfactory agreement with experiment, while significantly decreasing the computational cost. This workflow allows for cost efficient determination of energetic, thermodynamic and dynamic properties of WOCs.

## Graphical abstract

Water oxidation catalysts play a crucial role in the development of solar energy conversion devices. Thus, it is important to have computational tools that can reliably predict the catalytic mechanism and the energetics along the catalytic cycle. The approach proposed in this work combines a good accuracy with a small computational cost.

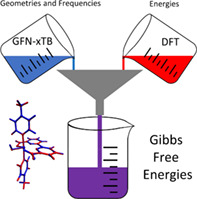

## 1 INTRODUCTION

Chemical fuels produced by solar energy have shown potential as a clean energy alternative to carbon‐based fossil fuels. Fuel production most commonly involves proton or CO_2_ reduction. The electrons needed for this reduction can be obtained through the oxidation of water into protons, molecular oxygen, and electrons. Therefore, water splitting dye‐sensitized photoelectrochemical cells and other photoelectrochemical devices for solar energy production have been intensely investigated in the recent decades.^1^, ^2^, ^3^ Still, the efficiency of such devices remains quite low as the water oxidation usually requires a high overpotential leading to significant energy losses. Although high overpotential can in principle be lowered by molecular catalysts, their operation with a sufficiently high turnover number and turnover frequency is a challenge. The development of a combination of a photosensitizer and a (transition metal‐based) stable and rapid catalyst in a photocatalytic complex is thus a crucial step forward in the realization of more efficient devices. Ru‐based transition metal complexes have in this respect in the last decade emerged as promising water oxidation catalyst (WOC) candidates.^4^, ^5^, ^6^, ^7^, ^8^

In addition to the catalyst itself, also the coupling of the WOC to a suitable photooxidative dye is challenging due to the different requirements for oxidation potentials and HOMO energies for the different catalytic steps that involve a number of oxidation states of the transition metal. Electronic configurations change through the catalytic cycle, which affects the nature of the HOMO and its energy. Computational investigations with for example, estimation of redox potentials, orbital energies, and excitation energies can help in finding suitable combinations of dye and WOC candidates.^9^ Dynamics and solvent effects should also be taken into account to simulate both water oxidation as well as the initial photooxidation of a dye‐WOC complex to obtain reliable insight in electron and hole transfer processes.^10^ DFT‐based ab initio molecular dynamics coupled with enhanced sampling techniques have been employed quite successfully in determining reaction barriers for water oxidation processes with oxidized WOCs,^11^, ^12^, ^13^, ^14^ redox mediators^15^, ^16^ or photo‐oxidized dyes.^17^, ^18^, ^19^, ^20^ However, these simulations are computationally very demanding as they require a quantum mechanical description of the entire system, including explicit solvation, since the water solvent participates actively in the catalytic reaction. Methods that well describe the organic molecular dyes, transition metal complexes and water are therefore needed, but this is complicated due to the open shell nature of the systems and the different spin states that need to be taken into account. Finding a computationally affordable and yet reliable method to determine thermodynamic requirements, oxidation potentials, and dynamic properties remains challenging.

A semiempirical method that has shown great potential in the structural characterization of transition metal complexes is the so called GFN‐xTB (Geometries, Frequencies, Noncovalent interactions extended Tight Binding) method developed by Grimme et al.^21^ This method is based on atomic and global parameters that are optimized on basis of DFT. GFN‐xTB has been used rather successfully for the description of transition metal complexes^21^, ^22^, ^23^ and lanthanoides.^24^ This method has been also extended on several fronts, including the so called GFN‐2xTB, that is founded on more physically relevant parameters and the GFN‐FF,^25^, ^26^ a force field parametrized on GFN‐xTB results. Both GFN‐xTB and GFN‐2xTB have already been used in the determination of redox potentials with low computational cost.^27^, ^28^, ^29^

In this study, we determine whether GFN‐xTB is a viable alternative to describe a Ru‐based catalyst developed by Duan et al.,^4^ also with respect to potential molecular dynamics simulations. Therefore, we investigated the performance of GFN‐xTB on this Ru‐based WOC in comparison to DFT with different exchange correlation functionals and to available experimental data. We propose a computationally efficient workflow that combines GFN‐xTB calculations for geometries and frequencies and B3LYP for energies, which leads to accurate relative Gibbs free energies along the catalytic cycle.

**SCHEME 1:**
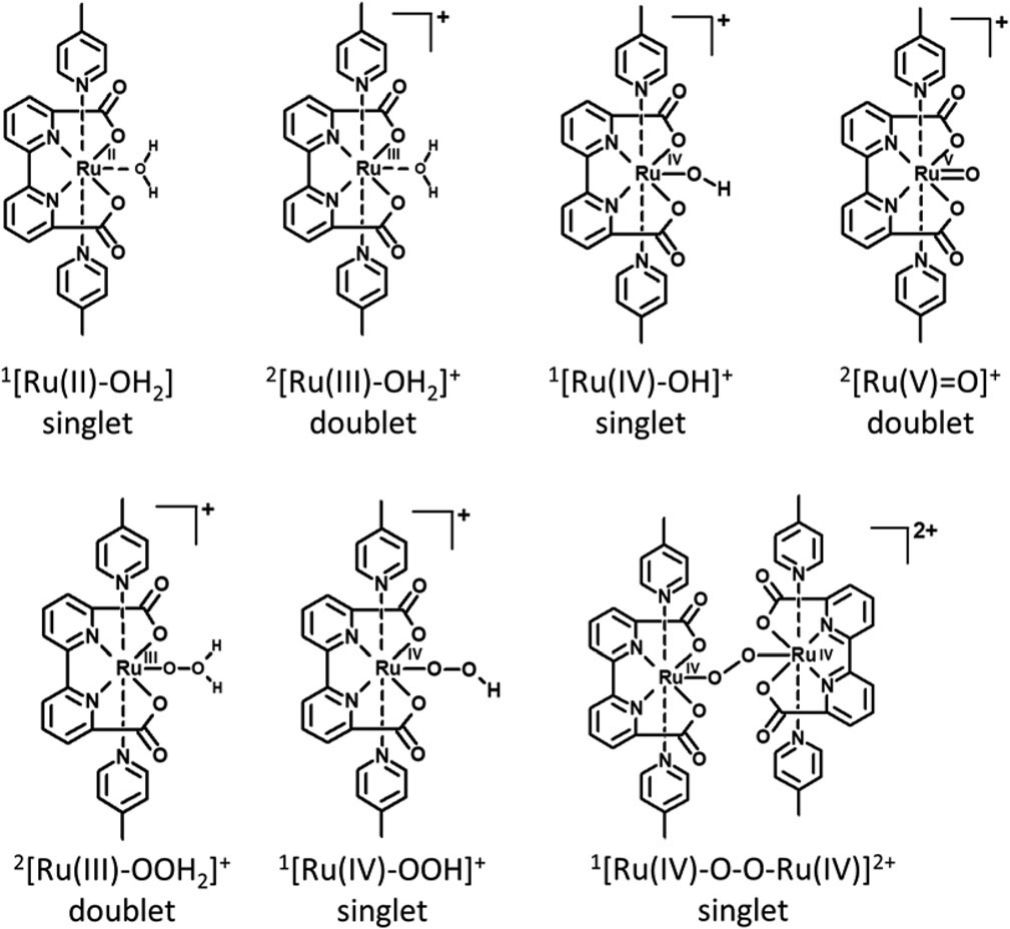
Chemical structures, abbreviations used throughout the publication and favored spin state of all catalytic intermediates

**SCHEME 2:**
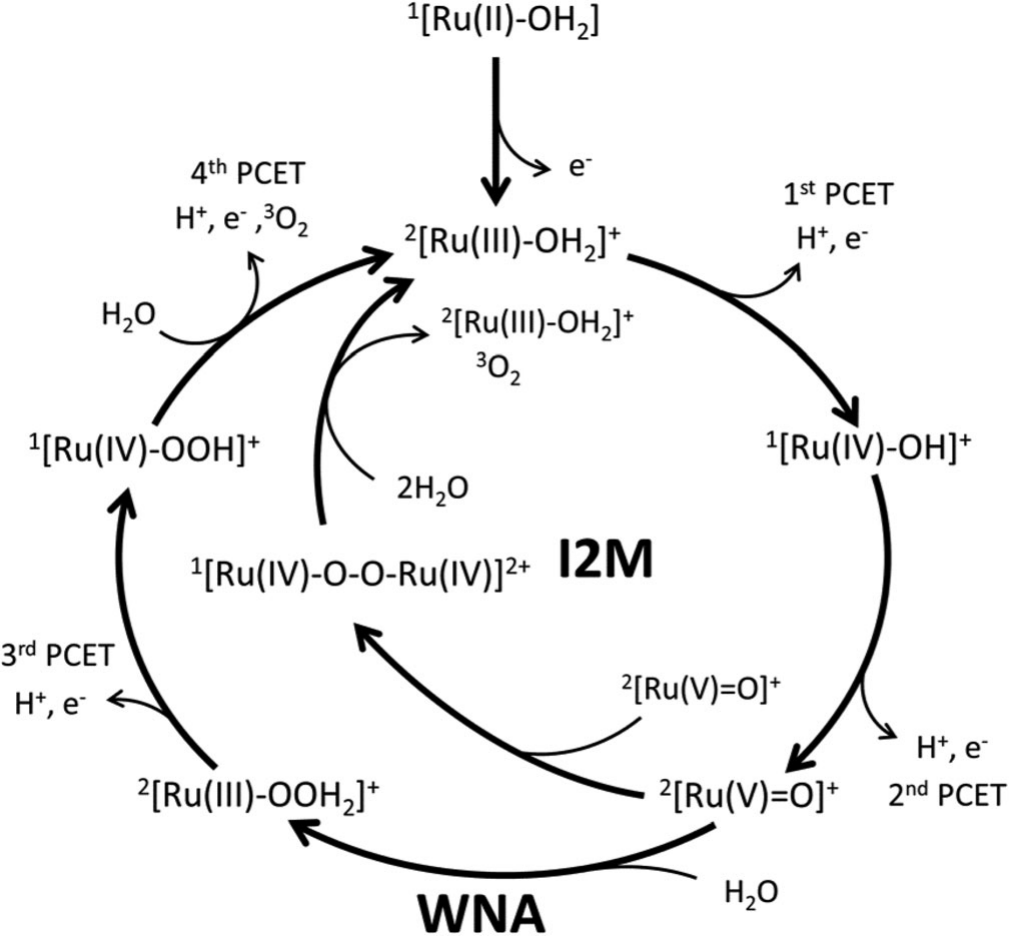
Catalytic cycle with the two possible pathways from ^2^[Ru(V) = O]^+^, water nucleophilic attack (WNA) and interaction of 2 metal oxo species (I2M)

The possible catalytic intermediates of the WOC are shown in Scheme 1, with their chemical structure, abbreviations used throughout the manuscript and their favored spin states. This class of catalysts has two possible catalytic pathways branching from the ^2^[Ru(V) = O]^+^ intermediate shown in Scheme 2. One is the water nucleophilic attack (WNA)^30^, ^31^ pathway, where a water molecule attacks the oxygen of the ^2^[Ru(V) = O]^+^ as a nucleophile, leading to the ^2^[Ru(III)‐OOH_2_]^+^.^30^ The other possible pathway involves two ^2^[Ru(V) = O]^+^ forming the ^1^[Ru(IV)‐O‐O‐Ru(IV)]^2+^ dimer through a radical coupling mechanism: this mechanism has been called interaction of two metal oxo species (I2M) or radical oxo coupling (ROC) in the literature.^32^, ^33^

This binuclear reaction pathway often shows higher turnover frequencies and lower overpotentials than the WNA pathway. It also circumvents the problem of scaling relations between the Ru‐OH and Ru‐OOH bond strength that makes optimizing catalysts far from trivial due to the interdependence of these two parameters.^34^, ^35^ However, the reaction rate in the I2M mechanism depends on the catalyst concentration, since the coupling involves two catalysts in the correct oxidation state, and can be accelerated by increasing the local concentration, for example, through accumulation in self‐assembling nanospheres.^36^

The investigated catalyst has been shown experimentally to perform via the I2M mechanism.^4^ However, by exchanging both equatorial or axial ligands, the catalyst can be tailored and the I2M mechanism can be made either more dominant or less favorable, to the point where the WNA mechanism is enforced over the I2M route.^37^, ^38^, ^39^, ^40^ In general, complexes with excess spin density on the oxygen and attractive interactions between axial ligands of two complexes lead to I2M mechanisms, whereas more positive partial charge on the same oxygen (and thus a higher electrophilicity) and steric hindrance between two complexes leads to a preferred WNA.^32^, ^38^, ^39^ Since both reaction mechanisms are possible for the investigated complex in different regimes, such as low concentration, immobilizing the catalyst on a surface and so forth, it is crucial that the computational methods used to investigate the catalyst describe both mechanisms reasonably well and predict the right mechanism. We therefore also evaluate the different methods with respect to predicting the correct catalytic pathway.

## 2 METHODS

### 2.1 Computational methods

All geometry optimizations as well as the vibrational analysis were performed with the Amsterdam Density Functional (ADF) and Density Functional Tight‐Binding (DFTB) engines of the Amsterdam Modeling Suite (AMS) program package.^41^, ^42^, ^43^ We decide to use structures including only the first coordination shell. Previous studies have also considered a small cluster of hydrogen bonded water molecules.^44^ However, the attachment of this tight water network results in considerable deformation of the original Ru complex, such as elongation of Ru‐N distances, deforming the bipyridine backbone. Moreover, these hydrogen bond networks remain stable only if the released protons are still attached to the complex without diffusing into bulk solution.

#### 2.1.1 DFT‐based simulations

Four different exchange correlation functionals were used in the DFT‐based investigations: B3LYP,^45^, ^46^ BLYP,^46^, ^47^ PBE,^48^, ^49^ and OPBE.^48^, ^49^, ^50^ All DFT‐based simulations were performed with the Slater type TZP (triple‐ζ polarized) basis set.^51^ D3 dispersion corrections with BJ‐damping were used.^52^ Scalar Relativistic effects were included via the Zero‐Order Regular Approximation (ZORA).^53^, ^54^, ^55^ The solvent environment was included through the COSMO implicit‐water model.^56^ Geometry optimizations were performed with unrestricted DFT, considering all possible spin states involving the d‐orbitals. Vibrational analysis was done only for the geometries corresponding to the most stable spin state.

#### 2.1.2 GFN‐xTB‐based simulations

For a semi‐empirical description of the Ru‐based water oxidation catalyst, the Geometry, Frequency and Noncovalent interaction Extended Tight Binding (GFN‐xTB) developed by Grimme et al. was used as implemented in the AMS2019 program package.^21^, ^43^ This was done to keep the same optimization and numerical frequency methods as for the DFT‐based calculations for a better direct comparison. Solvation was implicitly included via the Generalized Born accessible Surface Area (GBSA) model as implemented in AMS.^57^ Geometry optimizations were performed with fractional occupations corresponding to the different possible spin states, since unrestricted calculations are not supported yet.

#### 2.1.3 Thermodynamic computational investigations at pH = 0

The Gibbs free energy was determined using DFT with the four tested exchange correlation functionals mentioned above, as well as using GFN‐xTB. After optimization of the geometries of all catalytic intermediates in the lowest spin state and obtaining their binding energy (*E*), the zero‐point energy (*ZPE*), *pV* and entropic terms (*TS*) were estimated via vibrational analysis to determine the Gibbs free energy as given in Equation 1.

(1) $$G=E+\mathrm{pV}+\mathrm{ZPE}-TS_{\left(T=298.15\;K\right)}$$

This was also done for H_2_O, O_2_ and H_2_. Given the proton coupled electron transfer (PCET) character of the reaction steps, the free energy of H^+^ and e^−^ is computed as the free energy of ½ H_2_, as first proposed by Nørskov and co‐workers.^58^, ^59^, ^60^ The relative Gibbs free energy of each catalytic step is therefore taken as the difference between consecutive catalytic intermediates (including water, H_2_, and O_2_). Due to equating the free energy of a proton and electron with half hydrogen molecule, the Gibbs free energies are taken at standard NHE conditions. This means that all values, if not declared differently, are taken at pH = 0. The zero of the free energy is taken as the free energy of the first catalytic intermediate, the ^2^[Ru(III)‐OH_2_]^+^ plus two water molecules.

#### 2.1.4 Comparison to experimental oxidation potentials at pH = 1

To compare the computational results with experimental data, oxidation potentials for the first few catalytic steps were estimated from our Gibbs free energy calculations via Gibbs free energy difference between consecutive catalytic steps as first proposed and implemented by Nørskov and coworkers.^58^ Oxidation potentials were determined for the three oxidation reactions given in Equations (2), (3), (4), since for these steps there are experimental data available in the literature.^4^

(2) Ru(II)−OH21→Ru(III)−OH22++e−

(3) Ru(III)−OH22+→Ru(IV)−OH1++H++e−

(4) Ru(IV)−OH1+→Ru(V)=O2++H++e−

The first oxidation potential was determined by ΔSCF,^61^, ^62^ from the difference between the energy of the precatalyst ^1^[Ru(II)‐OH_2_] and its oxidized form ^2^[Ru(III)‐OH_2_]^+^ at the same geometry. Since this oxidation is pH independent and does only involve an electron transfer, the relaxation of the environment can be neglected as it is slow in comparison to the fast electron transfer. We also note that intake of water for the Ru(II) oxidation state is quite challenging and could in our model only be achieved by breaking a coordination bond between one carboxylic acid group and the ruthenium, with formation of a hydrogen bond between water and this carboxylate. A representative geometry is shown in Figure S1. This finding supports the suggestion that the complex is not stable in the 7‐coordinated state at this low oxidation state and water coordinates to the complex under breaking of one of the other coordination bonds to form a 6‐coordinated complex.^6^ Since the reaction involves the extraction of an electron without a coupled proton transfer, the energy difference is essentially determined against the absolute electrode potential (as in comparison to vacuum). Therefore, the energy needs to be converted to the normal hydrogen electrode (NHE) used in the experiment by subtracting the NHE potential energy versus vacuum (4.44 eV). This is also the only step, in which the number of electrons differs from the reactant to product states. GFN‐xTB has a particularly large self‐interaction energy that is corrected here by an empirical energy shift for the single point energy of the oxidized state in the ΔSCF by −5.70 eV as determined and employed by Neugebauer et al.^27^

The other two reaction steps involve a proton coupled electron transfer. Here, the reorganization of the complex is necessary for the oxidation to take place, especially the coupled proton transfer. Therefore, Gibbs free energy differences between the reactants and products are used to obtain the oxidation potentials. As mentioned earlier, the proton and electron release is assumed to be equivalent to the release of half a hydrogen molecule. These potentials are therefore already versus NHE at pH 0 since this corresponds to the energy needed to reduce a proton at standard conditions. As the experimental values were determined at pH = 1, the computed oxidation potentials were adjusted to this pH value by shifting the H^+^ potential using the Nernst equation as shown in Equation 5.

(5) $$G_{H_{}^{+},\mathrm{pH}=1}=G_{H_{}^{+},\mathrm{pH}=0}^{0}+k_{B}T\;*\;\ln\left(\left[H_{}^{+}\right]\right)=0-k_{B}T\ln\left(10\right)*{\mathrm{pH}}_{=1}^{}\approx -0.059\;\mathrm{eV}$$

here, $G_{H_{}^{+},\mathrm{pH}=0}^{0}$ is the standard Gibbs free energy at standard conditions with pH = 0, which is 0 eV by definition in NHE, *k_B_* the Boltzmann constant (~8.617 eV/K) and *T* is the temperature at standard conditions (*T* = 298,15 K). Following the Nernst equation for a single electron event, the computationally determined oxidation potentials involving a PCET step were shifted by −0.059 V to obtain the values versus NHE at pH = 1.

#### 2.1.5 GFN‐xTB + DFT approach for free‐energy calculations

For a good compromise between accuracy and computational cost, a combination of DFT and GFN‐xTB calculations was performed to estimate the Gibbs free energy. Since the geometries obtained at the GFN‐xTB and B3LYP level are remarkably similar (see Figure S2 and Table S6), geometry optimization and vibrational analysis can be done with GFN‐xTB, thereby significantly speeding up the process. The composite free energy consists of a DFT based energy to which the ZPE and entropic contributions obtained by GFN‐xTB are added (see Equation 6).

(6) $$G_{\mathrm{GFN}‐\mathrm{xTB}+\mathrm{DFT}}=E_{\mathrm{DFT}}^{\mathrm{GFN}‐\mathrm{xTB}\mathrm{geometry}}+{\mathrm{ZPE}}_{\mathrm{GFN}‐\mathrm{xTB}}+{\mathrm{pV}}_{\mathrm{GFN}‐\mathrm{xTB}}-{\mathrm{TS}}_{\mathrm{GFN}‐\mathrm{xTB}}$$

**SCHEME 3:**
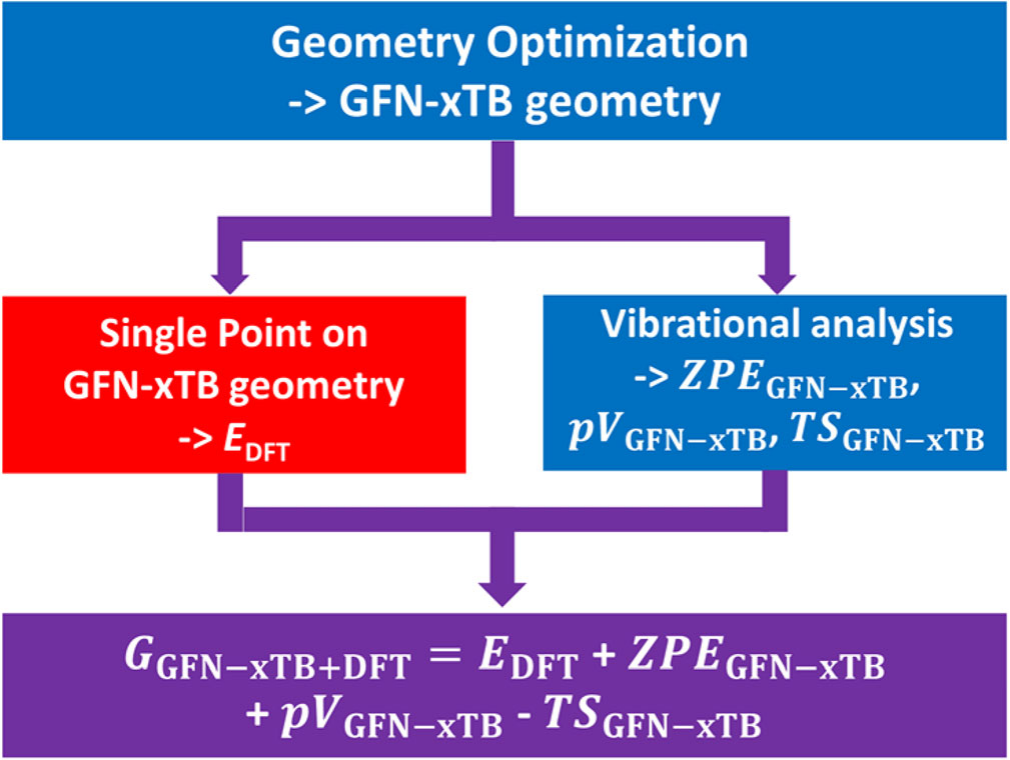
Workflow for the GFN‐xTB + DFT combined method. The colors denote the different computational methods used: Blue represents GFN‐xTB, red DFT. Purple denotes the combination of both. After a GFN‐xTB based geometry optimization, vibrational analysis with GFN‐xTB is performed, and the calculation is completed with a single point DFT at this geometry. The combination of these results gives an estimate of the Gibbs free energy (purple box)

The procedure is also visualized in Scheme 3. This workflow was tested using B3LYP, OPBE and the double hybrid functionals rev‐DOD‐PBE, rev‐DOD‐PBEP86, and rev‐DOD‐BLYP.^63^, ^64^, ^65^ The settings for the Double Hybrid calculations were the same as for the other DFT based simulations (thus including COSMO water, D3 [BJ] corrections, relativistic effects via ZORA), except for the higher basis set TZ2P.^51^, ^52^, ^53^, ^54^, ^55^, ^56^

We also note that in a very recent work by Spicher et al., the Single Point Hessian (SPH) method has been introduced, which combines GFN‐xTB/GFN‐FF methods with DFT to obtain reliable free energies in non‐equilibrium geometries, for example, obtained with a different level of theory or from molecular dynamics snapshots.^66^

## 3 RESULTS

### 3.1 Energetically preferred spin states

For all catalytic intermediates, the energetically favored state turned out to be the one with the lowest possible spin multiplicity. This is due to the 7‐coordinated environment of the Ru, breaking the octahedral symmetry and thus removing the t2g orbitals degeneracy. This result is consistently found using both GFN‐xTB and DFT with all exchange correlation functionals considered in this work (see Tables S1–S5). The corresponding favored spin state is reported in Scheme 1 for all intermediates. It should also be noted that for all cases, the states of higher multiplicity lie significantly higher in energy than the ground‐state (see Section S1) and are therefore neglected in further investigations. We note however, that an intersystem crossing (ISC) event is necessary for the release of oxygen from the dimer. Formation of the dimer is a radical coupling mechanism that necessitates two antiferromagnetically coupled ^2^[Ru(V) = O]^+^ species, since the formation of the new covalent bond is due to radical coupling of these two unpaired electrons. If the spins are parallel, thus ferromagnetically coupled, the bond formation has much less of a radical coupling character and thus requires a higher activation energy. This bond formation was studied computationally by Nyhlén et al., who also found a higher barrier for the ferromagnetically coupled ^2^[Ru(V) = O]^+^ pair.^44^ For the formation of ^3^O_2_, an ISC from singlet to triplet state is necessary. The authors showed that the dimer in the triplet state has a very low barrier toward oxygen release.^44^ In the WNA mechanism, the last step also necessitates an ISC for ^3^O_2_ release.

### 3.2 Relative Gibbs free energy along the catalytic cycle

**FIGURE 1:**
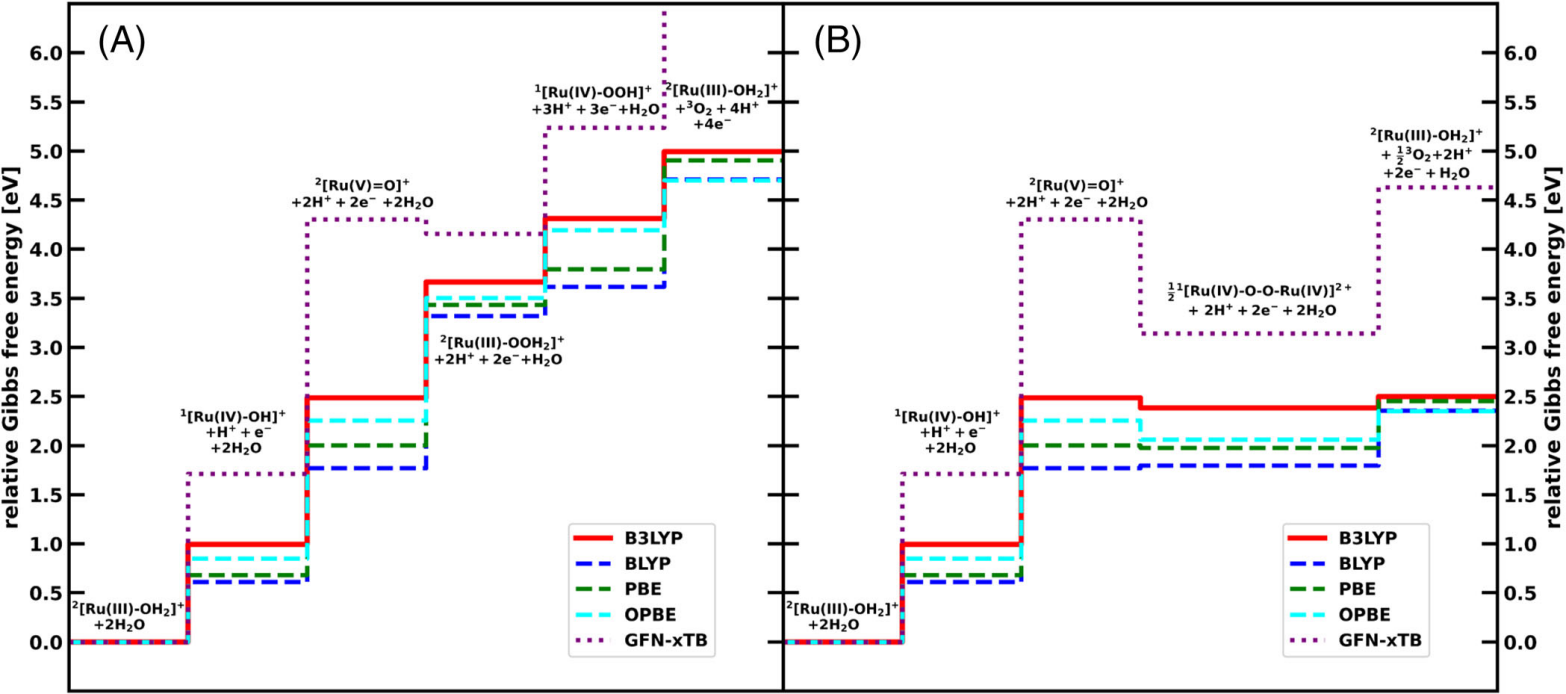
Gibbs free energies at pH = 0 relative to the first catalytic step along the catalytic pathways for (A) the WNA mechanism and (B) the I2M mechanism obtained with: B3LYP (red, solid line), BLYP (blue, dashed), PBE (green, dashed), OPBE (cyan, dashed), and GFN‐xTB (purple, dotted)

The Gibbs free energy differences of all catalytic intermediates at pH = 0 with respect to the first intermediate (^2^[Ru(III)‐OH_2_]^+^+2H_2_O) are given in Figure 1, as well as Table 1. The steps for the hybrid functional (B3LYP) are indicated with a solid line, the results for the GGA functionals (BLYP, PBE, OPBE) in dashed lines, while the GFN‐xTB trace is shown as a dotted line. With the exception of GFN‐xTB, all methods agree qualitatively with each other.

**TABLE 1:** Gibbs free energy difference in eV at pH = 0 for all catalytic steps and tested methods relative to the first catalytic intermediate

Method	^2^[Ru(III)‐OH_2_]^+^+2H_2_O	^1^[Ru(IV)‐OH]^+^+H^+^+e^−^ + 2H_2_O	^2^[Ru(V) = O]^+^+2H^+^+2e^−^+2H_2_O	^2^[Ru(III)‐OOH_2_]^+^+2H^+^+2e^−^+H_2_O	^1^[Ru(IV)‐OOH]^+^+3H^+^+3e^−^+H_2_O	½^1^[Ru(IV)‐O‐O‐Ru(IV)]^2+^+2H^+^+2e^−^	^2^[Ru(III)‐OH_2_]^+^+4H^+^+4e^−^+^3^O_2_
B3LYP	0.00	0.99	2.49	3.67	4.31	2.38	5.00
BLYP	0.00	0.61	1.77	3.32	3.62	1.80	4.71
PBE	0.00	0.68	2.00	3.43	3.80	1.98	4.91
OPBE	0.00	0.85	2.25	3.50	4.19	2.06	4.70
GFN‐xTB	0.00	1.71	4.30	4.16	5.24	3.14	9.26
GFN‐xTB+ B3LYP	0.00	0.99	2.57	3.93	4.45	2.49	5.12
GFN‐xTB+ OPBE	0.00	1.01	2.46	3.83	4.41	2.38	4.82

For the WNA pathway, all catalytic steps are endergonic for all methods with the exception of GFN‐xTB, that gives a slightly exergonic nucleophilic attack step. In general, the Gibbs free energy obtained with GFN‐xTB deviates significantly from the other methods, with for example the oxygen release step 4.26 eV higher than the closest DFT value, obtained with B3LYP. The large energetic difference is most likely due to use of the fractional occupations instead of a properly unrestricted calculation of the oxygen molecule using GFN‐xTB. This choice of occupations leads to energies that are always higher than obtained for a closed shell singlet state. Therefore, the formation energy of molecular oxygen is overestimated. Also for the ^2^[RuV=O]^+^ intermediate, GFN‐xTB gives a free energy 1.81 eV higher than the next closest method (B3LYP).

The conclusion from these results is that GFN‐xTB in the present form (not allowing for unrestricted calculations) appears to be unsuitable for correctly describing the energetic and thermodynamic properties of the different catalytic intermediates. We note that this is not surprising, since GFN‐xTB has been developed with the goal of computing accurate geometries and frequencies, but not primarily for accurate energetics.

### 3.3 Preferred reaction mechanism

Experimental investigations on the catalyst showed that it operates via the I2M radical coupling mechanism.^4^ All investigated methods predict this correctly. As visible in Figure 1(B), the radical coupling between two ^2^[Ru(V) = O]^+^ species leads to the energetically lower ^1^[Ru(IV)‐O‐O‐Ru(IV)]^2+^ dimer species. This downhill process is in contrast with the thermodynamically unfavorable formation of ^2^[Ru(III)‐OOH_2_]^+^ via water nucleophilic attack. The only exception to this is GFN‐xTB, where both processes, I2M as well as WNA, are downhill. However, the free energy difference between ^2^[Ru(V) = O]^+^ and the dimer is much larger than between ^2^[Ru(V) = O]^+^ and ^2^[Ru(III)‐OOH_2_]^+^, also predicting the I2M mechanism to be much more likely. All methods show the oxygen release from the dimer to be endergonic under standard conditions. However, we note that this endergonic behavior might be an artifact of the missing explicit water solvation, as the water uptake is highly sensitive to the hydrogen bonding network.^44^ Furthermore, an ISC event is necessary before triplet oxygen can be released. The triplet state of the dimer, as seen in the SI, is considerably higher in energy than the singlet for all investigated methods, making the oxygen release favorable and irreversible. While for the DFT based methods, the Gibbs free energy difference between the dimer and the final catalytic step to regenerate the ^2^[Ru(III)‐OH_2_]^+^ under the release of oxygen is relatively small, it is quite large for GFN‐xTB. Although this does not change the preference for the I2M mechanism, it underlines again that GFN‐xTB alone is not suited to describe the energetics in the catalytic cycle in a satisfactory manner.

### 3.4 Relative Gibbs free energy using GFN‐xTB + DFT

**FIGURE 2:**
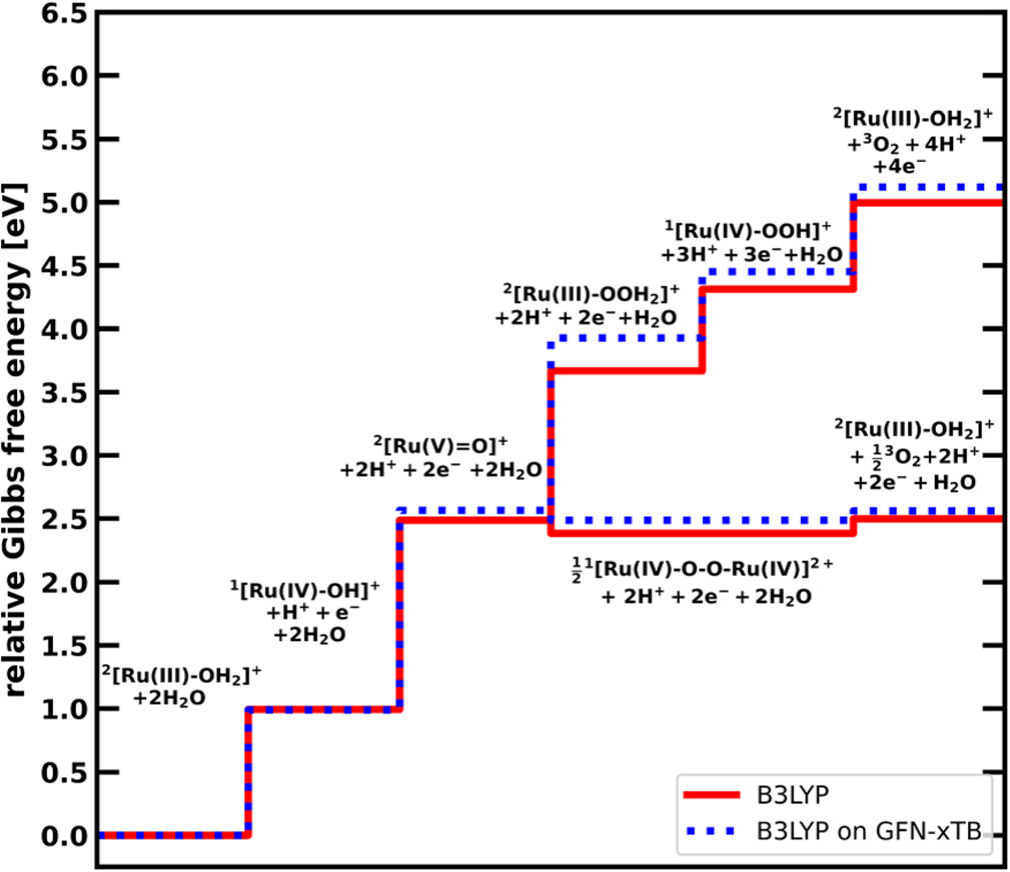
Gibbs free energies at pH = 0 relative to the first catalytic step along both catalytic pathways obtained by B3LYP (red, solid line) and by the GFN‐xTB + B3LYP combined approach (blue, dotted line)

While GFN‐xTB did not prove to be reliable for the energies, it provides geometries and frequencies that are quite similar to the B3LYP results (see root mean square displacements and the Internal Energy and entropic terms in Table S6). For this reason, a combination of GFN‐xTB geometries and frequencies with single point energies from B3LYP were also tested (see Scheme 3). Using a geometry optimization and vibrational analysis from GFN‐xTB in combination with a single point energy from B3LYP significantly reduced computational cost while still providing reliable results for the free energy. The relative Gibbs free energy of the catalytic intermediates for both the WNA and the I2M mechanism is given in Figure 2 and Table 1 for both B3LYP and GFN‐xTB + B3LYP. As can be seen in Figure 2, the GFN‐xTB + B3LYP method predicts the correct reaction mechanism and follows very closely the B3LYP results: the deviation from B3LYP is quite small, around 0.1 eV for all the steps except one deviating by 0.26 eV. While the energy differences between consecutive steps are in excellent agreement, the accumulation of small errors lead to larger deviations for the later intermediates, as they are taken relative to the first step. The relatively large structural difference for the ^2^[Ru(III)‐OOH_2_]^+^ intermediate (see Table S6) between the two methods might explain the slightly larger deviation at this step.

While this combination of B3LYP and GFN‐xTB gives almost quantitatively the same results as the B3LYP, the computational cost is drastically reduced as only one single point calculation with the hybrid functional is needed, thus avoiding the demanding geometry optimization, and more importantly, frequency calculation. As the computational cost for GFN‐xTB is extremely small compared to B3LYP (see Table S6), this leads to a speed up of a minimum of 2 orders of magnitude for this system, as 2*3 N (>300) points need to be calculated for the numerical differentiation of the analytical gradient of the energy in the frequency analysis.

The extreme speed of GFN‐xTB leads to the possibility of describing large, extended systems for the cost of essentially only one single point calculation of a higher‐level method such as B3LYP, with this calculation being the limiting factor. This combined method therefore effectively scales as a single point calculation of the higher‐level method. For systems that can be treated with a single point DFT calculation, the combined method allows for accurate Gibbs free energies with little additional cost.

The semi‐quantitative agreement with a much lower computational cost also holds true when using OPBE for the single point energies (Table 1, last row).

It is quite remarkable that GFN‐xTB describes the geometries and frequencies of these challenging transition metal complexes so well, especially considering the different oxidation and spin states involved. Furthermore, although GFN‐xTB was not developed for this purpose, it can be extended by just one additional single point calculation based on DFT to give remarkably accurate Gibbs free energies.

All in all, this combination of DFT and GFN‐xTB shows great potential in obtaining results close to the DFT description while being much more efficient computationally.

### 3.5 Comparison to experimental oxidation potentials

**FIGURE 3:**
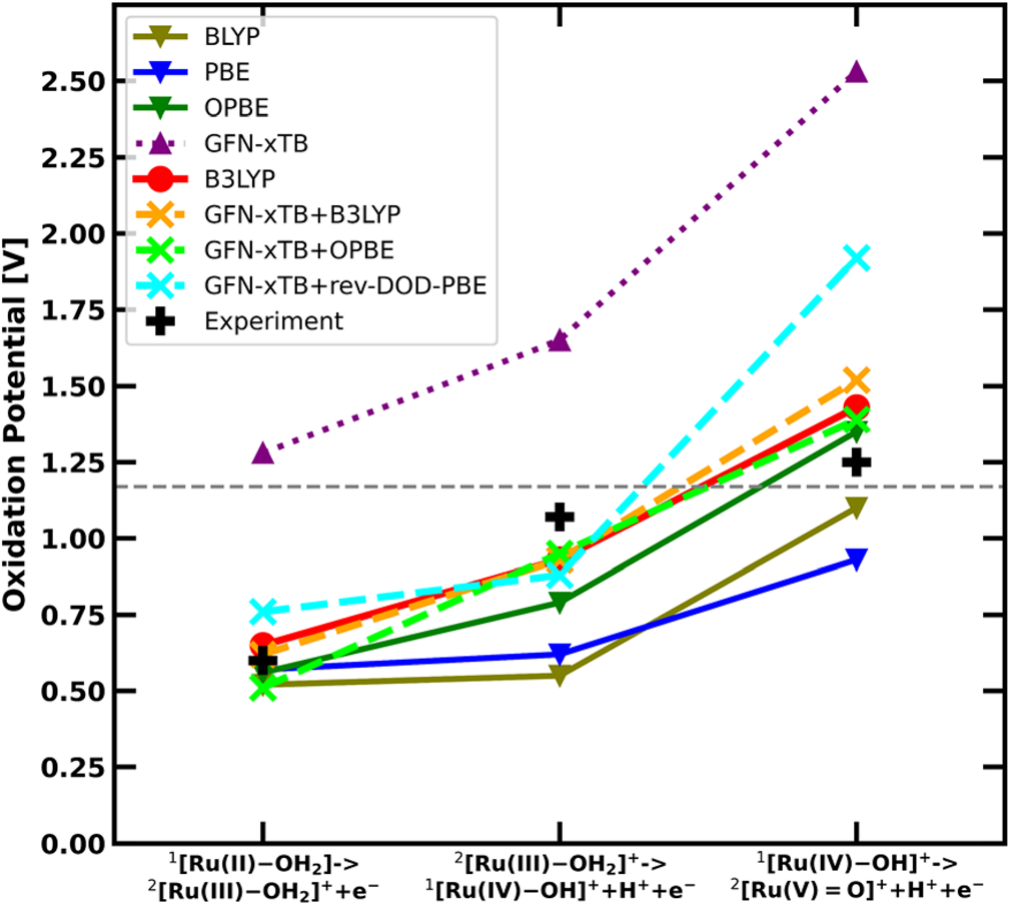
Computed oxidation potentials versus NHE at pH = 1 between catalytic steps in comparison to experimental data (black crosses): B3LYP (red circles), BLYP (olive triangles), PBE (blue triangles), OPBE (green triangles), GFN‐xTB (purple triangles), GFN‐xTB + B3LYP (orange crosses), GFN‐xTB + OPBE (lime crosses) and the double hybrid rev‐DOD‐PBE with GFN‐xTB geometry and frequencies (cyan crosses). The lines connecting the symbols are merely to guide the eye. The gray dotted line represents the optimal oxidation potential of water at pH = 1

The experimentally determined oxidation potential of the ^1^[Ru(II)‐OH_2_] ‐ > ^2^[Ru(III)‐OH_2_]^+^, ^2^[Ru(III)‐OH_2_]^+^ − > ^1^[Ru(IV)‐OH]^+^, and ^1^[Ru(IV)‐OH]^+^ − > ^2^[Ru(V) = O]^+^ steps are given in Table 2 and compared to the calculated oxidation potentials versus NHE at pH = 1 for all investigated methods. The results are also shown graphically in Figure 3.

**TABLE 2:** Oxidation potentials in V versus NHE at pH = 1 for the given oxidative steps obtained for all used computational methods and experimental values

Method	^1^[Ru(II)‐OH_2_] → ^2^[Ru(III)‐OH_2_]^+^ + e^−^	^2^[Ru(III)‐OH_2_]^+^ → ^1^[Ru(IV)‐OH]^+^ + H^+^ + e^−^	^1^[Ru(IV)‐OH]^+^ → ^2^[Ru(V) = O]^+^ + H^+^ + e^−^
B3LYP	0.65	0.93	1.43
BLYP	0.52	0.55	1.10
PBE	0.57	0.62	0.93
OPBE	0.56	0.79	1.35
GFN‐xTB	1.28^a^	1.65	2.53
GFN‐xTB + B3LYP	0.62	0.93	1.52
GFN‐xTB + OPBE	0.51	0.95	1.39
GFN‐xTB + rev‐DOD‐PBE	0.76	0.88	1.92
GFN‐xTB + rev‐DOD‐PBEP86	0.73	0.91	1.92
GFN‐xTB + rev‐DOD‐BLYP	0.80	0.88	2.03
Experiment	0.60^b^	1.07^b^	1.25^b^

^a^ Including self‐interaction correction.^27^

^b^ From reference.^4^

In addition to the results reported in Table 1, also single point energies of three double hybrid functionals, rev‐DOD‐PBE, rev‐DOD‐PBEP86, and rev‐DOD‐BLYP in combination with GFN‐xTB geometries and frequencies are included here. All methods show the same trend for the three oxidative steps, with an increasing oxidation potential.

The B3LYP and the OPBE results are in good agreement with experimental values, the largest absolute deviation from experimental results being 0.18 V for B3LYP and 0.28 V for OPBE. Interestingly, our results using B3LYP are very close to the results obtained in Reference 44, even though the model used and the approach to compute the free energy were different. PBE and BLYP deviate more, with a maximum error of 0.52 V (BLYP) and 0.45 V (PBE). The cheapest method, GFN‐xTB shows a reasonable qualitative agreement but a significant overestimation of the oxidation potential for all reaction steps. The largest deviation from experiment is 1.28 V. While this shows that GFN‐xTB is not reliable enough on its own, the results of the GFN‐xTB + DFT combined methods show the reliability of geometries and frequencies of GFN‐xTB: when using those in combination with single point energies of B3LYP and OPBE, the difference to the full DFT methods is small. For GFN‐xTB + B3LYP, these differences range from 0.00 to 0.09 V versus the B3LYP, or a maximum of 0.27 V versus experiment. GFN‐xTB + OPBE has an absolute deviation by a maximum of 0.16 V versus OPBE and a maximum of 0.14 V when compared to experimental values. These values show that the combined GFN‐xTB + DFT methods give significantly more reliable results than some of the full DFT based methods as BLYP and PBE. We also tested some double hybrid functionals, as the need for only a single point calculation in this method makes them computationally accessible. While all three tested double hybrid functionals agree very well with another, they all significantly overestimate the oxidation potential for the oxidation to the ^2^[Ru(V) = O]^+^ species. The deviation here ranges from 0.67 V (rev‐DOD‐PBE and rev‐DOD‐PBEP86) to 0.78 V (rev‐DOD‐BLYP). In contrast, the agreement with the other two oxidative steps is quite good. Since only one of the steps shows such a large deviation, the oxidation potential was also determined using B3LYP geometries and frequencies combined with rev‐DOD‐PBE single point energies. Here, the deviation remained over 0.7 V as well (see Table S7). The poor performance of the double hybrids, in this case, is most likely due to the fact that they are used on geometries not optimized at the same level. Especially the ^2^[Ru(V) = O]^+^ geometry is quite deformed due to the high number of coordinated ligands and the double bond between ruthenium and oxygen. B3LYP geometries perform as poorly as GFN‐xTB. A more in‐depth analysis of double hybrid performance and their sensitivity with regards to geometries would be interesting for future investigations.

The good performance of GFN‐xTB with regards to geometries and frequencies could also provide a cheap method to investigate the effect of explicit solvent or other embedding environments on the thermodynamical properties. MD‐based equilibrations of the involved reactants and products in full solvation at the GFN‐xTB level can be followed by a vibrational analysis of selected snapshots, with a single point analysis on a DFT‐basis for reliable Gibbs free energy differences in a fully solvated system.

All in all, the combination of GFN‐xTB with higher level methods for a better description of the electronic structure seems quite reliable with a significant reduction of computational cost.

## 4 CONCLUSIONS

The full catalytic cycle of a ruthenium water oxidation catalyst was investigated using a wide range of DFT based methods as well as the tight binding approach GFN‐xTB. While all tested computational methods predict the correct spin state and catalytic pathway, GFN‐xTB fails to qualitatively describe the relative Gibbs free energy between the different catalytic steps, which is not surprising as this method was not developed to fulfill this purpose. B3LYP and OPBE both show the best performance in comparison to experiment, giving very similar results with an error of < 0.2/0.3 eV respectively. The other GGAs describe the process qualitatively correct but show larger deviations from experiment. As GFN‐xTB provides excellent geometries and frequencies, a computational workflow that combines GFN‐xTB geometries and frequencies with B3LYP single point energies is proposed and is found to closely reproduce relative Gibbs free energies of full B3LYP calculations while being at least 2 orders of magnitude faster than B3LYP. The same holds true when using OPBE. Usage of double hybrid functionals on the GFN‐xTB geometries is computationally highly accessible, but while the results show good agreement with experiment for the first two oxidation steps (within 0.2 V), the last oxidative step shows larger deviation. The combination of GFN‐xTB derived geometries and frequencies with higher level methods for a good electronic description of the WOC system shows great promise for fast, reliable determination of redox potentials, thermodynamic properties and electronic structure at a reasonable computational cost. The good description of both geometries and frequencies by GFN‐xTB should allow for computationally accessible molecular dynamics simulations and embedding in extensive simulation boxes including potential photosensitizers or redox mediators and explicit solvation.

## Supporting information

**Appendix S1**. Supporting Information.
