# Supplementary material for: Efficient workflow for the investigation of the catalytic cycle of water oxidation catalysts: Combining GFN‐xTB and density functional theory
Source: J Comput Chem. 2021 Jul 18;42(26):1885–94. doi: 10.1002/jcc.26721 (PMC8456855; doi:10.1002/jcc.26721)
Supplement: Supplementary file 1 — Appendix S1. Supporting Information. [file JCC-42-1885-s001.pdf]

Supporting Information for the Manuscript:

Efficient Workflow for the Investigation of the Catalytic Cycle of Water Oxidation Catalysts: Combining GFN-xTB and DFT

Jan Paul Menzel†, Martijn Kloppenburg†, Jelena Belić‡, Huub J.M. de Groot†, Lucas Visscher‡, Francesco Buda†**

† Leiden Institute of Chemistry, Leiden University, PO Box 9502, 2300 RA Leiden, The Netherlands.

‡ Department of Chemistry and Pharmaceutical Sciences, Vrije Uversiteit Amsterdam, De Boelelaan 1083, Amsterdam 1081 HV, The Netherlands.

Corresponding Authors

*E-mail: f.buda@lic.leidenuniv.nl, j.p.menzel@lic.leidenuniv.nl

SI-1 energetically preferred spin states

SI-2 6-coordinated ^{1}[Ru(II)-OH$_2$]

SI-3 Energy Terms in Gibbs Free Energy and Computational Cost

SI-4 Double Hybrid on B3LYP geometries

SI-1 Energetically preferred spin states

All DFT based calculations were performed with the ADF2019[1,2] program by SCM using a TZP basis set,[3] D3 dispersion corrections with BJ-damping,[4] and COSMO implicit water[5]. Relativistic effects were included via the ZORA model.[6–8] The investigated exchange correlation functionals are B3LYP,[9,10] BLYP,[10,11] PBE[12,13] and OPBE[12–14]. The GFN-xTB[15] calculations were performed by the DFTB package included in AMS2019[16] by SCM, including GBSA implicit water as implemented in AMS.[17]

Table S1. *Energies in eV of viable spin states for all catalytic intermediates using B3LYP. The most stable spin state is marked in bold.*

Intermediate	Singlet	Doublet	Triplet	Quartet	Quintet	Sextet
[Ru(II)-OH$_2$]	**-430.06**	-	-428.94	-	-427.90	-
[Ru(III)-OH$_2$]$^+$	-	**-425.48**	-	-425.31	-	-423.26
[Ru(IV)-OH]$^+$	**-420.39**	-	-419.81	-	-418.38	-
[Ru(V)=O]$^+$	-	**-414.73**	-	-413.62	-	-
[Ru(III)-OOH$_2$]$^+$	-	**-431.26**	-	-429.99	-	-428.65
[Ru(IV)-OOH]$^+$	**-426.56**	-	-426.11	-	-424.92	-
[Ru(IV)-O-O-Ru(IV)]$^{2+}$	**-830.48**	-	-829.72	-	-828.92	-
H_2	**-7.71**	-	-	-	-	-
O_2	-10.77	**-**	**-13.41**	**-**	**-**	**-**
H_2O	**-17.25**	-	-	-	-	-

Table S2. *Energies in eV of viable spin states for all catalytic intermediates using BLYP. The most stable spin state is marked in bold.*

Intermediate	Singlet	Doublet	Triplet	Quartet	Quintet	Sextet
[Ru(II)-OH$_2$]	**-358.61**	-	-357.314	-	-355.94	-
[Ru(III)-OH$_2$]$^+$	-	**-354.14**	-	-352.92	-	-351.49
[Ru(IV)-OH]$^+$	**-349.98**	-	-349.24	-	-347.78	-
[Ru(V)=O]$^+$	-	**-345.19**	-	-344.09	-	-
[Ru(III)-OOH$_2$]$^+$	-	**-358.01**	-	-356.60	-	-355.80
[Ru(IV)-OOH]$^+$	**-354.20**	-	-353.44	-	-352.20	-
[Ru(IV)-O-O-Ru(IV)]$^{2+}$	**-691.14**	-	-690.23	-	-689.38	-
H_2	**-6.65**	-	-	-	-	-
O_2	-8.11	**-**	**-9.24**	**-**	**-**	**-**
H_2O	**-13.95**	-	-	-	-	-

Table S3. *Energies in eV of viable spin states for all catalytic intermediates using PBE. The most stable spin state is marked in bold.*

Intermediate	Singlet	Doublet	Triplet	Quartet	Quintet	Sextet
[Ru(II)-OH$_2$]	**-375.11**	-	-373.66	-	-372.24	-
[Ru(III)-OH$_2$]$^+$	-	**-370.52**	-	-369.04	-	-367.65
[Ru(IV)-OH]$^+$	**-366.25**	-	-365.42	-	-364.90	-
[Ru(V)=O]$^+$	-	**-361.25**	-	-360.09	-	-
[Ru(III)-OOH$_2$]$^+$	-	**-374.67**	-	-373.06	-	-371.46
[Ru(IV)-OOH]$^+$	**-370.71**	-	-369.98	-	368.61	-
[Ru(IV)-O-O-Ru(IV)]$^{2+}$	**-723.32**	-	-722.33	-	-721.48	-
H_2	**-6.75**	-	-	-	-	-
O_2	-8.59	**-**	**-9.72**	**-**	**-**	**-**
H_2O	**-14.39**	-	-	-	-	-

Table S4. *Energies in eV of viable spin states for all catalytic intermediates using OPBE. The most stable spin state is marked in bold.*

Intermediate	Singlet	Doublet	Triplet	Quartet	Quintet	Sextet
[Ru(II)-OH_2]	**-383.01**	-	-381.74 Water released	-	-380.05 Water released	-
[Ru(III)-OH_2]$^+$	-	**-378.43**	-	-376.73	-	-357.57
[Ru(IV)-OH]$^+$	**-373.99**	-	-373.11	-	-371.48	-
[Ru(V)=O]$^+$	-	**-368.88**	-	-367.86	-	-
[Ru(III)-OOH_2]$^+$	-	**-382.64**	-	-376.73	-	-375.57
[Ru(IV)-OOH]$^+$	**-378.35**	-	-377.91	-	-376.15	-
[Ru(IV)-O-O-Ru(IV)]$^{2+}$	**-738.92**	-	-738.73	-	-738.80	-
H_2	**-6.79**	-	-	-	-	-
O_2	-8.89	-	**-10.19**	-	-	-
H_2O	**-14.58**	-	-	-	-	-

Table S5. *Energies in eV of viable spin states for all catalytic intermediates using GFN-xTB. The most stable spin state is marked in bold.*

Intermediate	Singlet	Doublet	Triplet	Quartet	Quintet	Sextet
[Ru(II)-OH_2]	**-2783.80**	-	-2782.48	-	-2780.47	-
[Ru(III)-OH_2]$^+$	-	**-2773.21**	-	-2771.59	-	-2768.75
[Ru(IV)-OH]$^+$	**-2757.21**	-	-2756.32	-	-2754.19	-
[Ru(V)=O]$^+$	-	**-2740.29**	-	-2738.94	-	-
[Ru(III)-OOH_2]$^+$	-	**-2897.88**	-	-2896.02	-	-2893.37
[Ru(IV)-OOH]$^+$	**-2882.50**	-	-2881.01	-	-2879.50	-
[Ru(IV)-O-O-Ru(IV)]$^{2+}$	**-5483.77**	-	-5482.93	-	-5482.02	-
H_2	**-28.15**	-	-	-	-	-
O_2	**-247.77**	-	**-247.77**	-	-	-
H_2O	**-156.935**	-	-	-	-	-

SI-2 6-coordinated ^{1}[Ru(II)-OH_2]

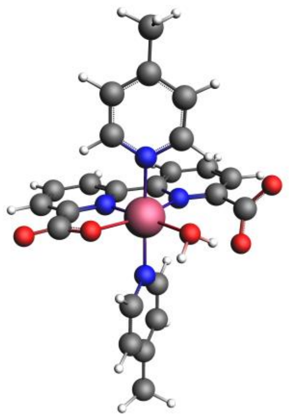

Figure S1. *Geometry of the ^{1}[Ru(II)-OH_2] complex. Note the breaking of the Ru-O bond from the carboxylate and hydrogen bond towards the bound water molecule to form a 6-coordinated complex.*

SI-3 Energy Terms in Gibbs Free Energy and Computational Cost

Table S6. *Energy decomposition of the different terms in the Gibbs free energy per catalytic intermediate for B3LYP and GFN-xTB. Also given is the computational cost for the vibrational analysis calculated on nodes with 24 cores. Root mean square displacements between GFN-xTB and B3LYP are given for the bond distances in the first coordination sphere.*

Intermediate / Method	Bond Energy [eV]	Internal Energy [eV]	pV term [eV]	-TS [eV]	Gibbs Free Energy [eV]	Calculation time (*Nr. of nodes) [min]	RMSD [Å]
$^{1}[Ru(II)\text{-}OH_2]$							
B3LYP	-430,06	12,44	0.03	-2.63	-420.23	868(*4=3472)	0.052
GFN-xTB	-2783.80	12.06	0.03	-2.70	-2774.40	1	
$^{2}[Ru(III)\text{-}OH_2]^{+}$							
B3LYP	-425.48	12.47	0.03	-2.64	-415.63	1300(*4=5200)	0.248
GFN-xTB	-2773.21	12.09	0.03	-2.82	-2763.91	3	
$^{1}[Ru(IV)\text{-}OH]^{+}$							
B3LYP	-420.39	12.17	0.03	-2.57	-410.76	1243(*4=4972)	0.041
GFN-xTB	-2757.21	11.80	0.03	-2.73	-2748.12	1	
$^{2}[Ru(V)=O]^{+}$							
B3LYP	-414.74	11.85	0.03	-2.54	-405.39	1899(*4=7596)	0.061
GFN-xTB	-2740.29	11.46	0.03	-2.66	-2731.46	1	
$^{2}[Ru(III)\text{-}OOH_2]^{+}$							
B3LYP	-431.26	12.60	0.03	-2.74	-421.37	2200(*4=8800)	0.122
GFN-xTB	-2888.47	12.25	0.03	-2.86	-2888.47	1	
$^{1}[Ru(IV)\text{-}OOH]^{+}$							
B3LYP	-426.56	12.30	0.03	-2.62	-416.85	1484(*4=5936)	0.031
GFN-xTB	-2882.50	11.94	0.03	-2.78	-2873.31	1	
$^{1}[Ru(IV)\text{-}O\text{-}O\text{-}Ru(IV)]^{2+}$							
B3LYP	-830.48	23.82	0.03	-4,36	-810.99	16893(*2=33786)	0.036
GFN-xTB	-5483.76	23.06	0.03	-4.54	-5465.23	3	
H_2							
B3LYP	-7.71	0.34	0.03	-0.40	-7.75	1	0.011
GFN-xTB	-28.15	0.38	0.03	-0.40	-28.15	<<1	
O_2							
B3LYP	-13.41	0.16	0.03	-0.61	-13.83	1	0.006
GFN-xTB	-247.77	0.17	0.03	-0.61	-248.17	<<1	
H_2O							
B3LYP	-17.25	0.64	0.03	-0.58	-17.16	1	0.014
GFN-xTB	-156.94	0.62	0.03	-0.58	-156.87	<<1	

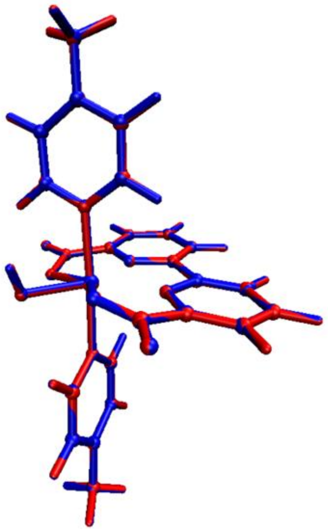

Figure S2. *Overlay of the molecular structures of the ^{1}[Ru(IV) OH]$^{+}$ molecule optimized with the semi-empirical GFN-xTB (blue) and DFT using the B3LYP exchange correlation functional (red).*

SI-4 Double Hybrids on GFN-xTB and B3LYP geometries

Double Hybrid Single Point calculations were performed using the same settings as described above (ZORA, COSMO, D3(BJ)) with the exception of using the higher TZ2P basis set. Calculations were performed with rev-DOD-PBE, rev-DOD-PBEP86 and rev-DOD-BLYP.[18,19] Single Point calculations were performed on GFN-xTB geometries. For comparison, a rev-DOD-PBE Single Point calculation was also done using geometries and frequencies obtained with B3LYP.

Table S7: *Determined Oxidation potentials compared to experiment at pH=1 for the three investigated Double Hybrid Functionals rev-DOD-PBE, rev-DOD-PBEP86 and rev-DOD-BLYP using COSMO and D3(BJ) on geometries obtained with GFN-xTB. Also added are determined Oxidation Potentials when using B3LYP geometries for the Double Hybrid rev-DOD-PBE.*

**Method**	**[Ru(II)-OH$_2$] -> [Ru(III)-OH$_2$]$^+$**	**[Ru(III)-OH$_2$]$^+$ -> [Ru(IV)-OH]$^+$**	**[Ru(IV)-OH]$^+$ -> [Ru(V)=O]$^+$**
Rev-DOD-PBE on GFN-xTB geometry	0.76 V	0.88 V	1.92 V
Rev-DOD-PBEP86 on GFN-xTB geometry	0.73 V	0.91 V	1.92 V
Rev DOD-BLYP on GFN-xTB geometry	0.80 V	0.88 V	2.03 V
Rev-DOD-PBE on B3LYP geometry	0.79 V	0.77 V	1.98 V
**Experiment**	**0.60 V**	**1.07 V**	**1.25 V**

References

1 G. te Velde, F. M. Bickelhaupt, E. J. Baerends, C. Fonseca Guerra, S. J. A. van Gisbergen, J. G. Snijders and T. Ziegler, *J. Comput. Chem.*, 2001, **22**, 931–967.

2 ADF 2019.3, SCM, Theoretical Chemistry, Vrije Universiteit, Amsterdam, The Netherlands, http://www.scm.com, .

3 E. Van Lenthe and E. J. Baerends, *J. Comput. Chem.*, 2003, **24**, 1142–1156.

4 S. Grimme, S. Ehrlich and L. Goerigk, *J. Comput. Chem.*, 2011, **32**, 1456–1465.

5 C. C. Pye and T. Ziegler, *Theor. Chem. Acc.*, 1999, **101**, 396–408.

6 E. van Lenthe, E. J. Baerends and J. G. Snijders, *J. Chem. Phys.*, 1993, **99**, 4597–4610.

7 E. van Lenthe, E. J. Baerends and J. G. Snijders, *J. Chem. Phys.*, 1994, **101**, 9783–9792.

8 E. van Lenthe, A. Ehlers and E.-J. Baerends, *J. Chem. Phys.*, 1999, **110**, 8943–8953.

9 A. D. Becke, *J. Chem. Phys.*, 1993, **98**, 1372–1377.

10 A. D. Becke, *Phys. Rev. A*, 1988, **38**, 3098–3100.

11 C. Lee, W. Yang and R. G. Parr, *Phys. Rev. B*, 1988, **37**, 785–789.

12 J. P. Perdew, K. Burke and M. Ernzerhof, *Phys. Rev. Lett.*, 1996, **77**, 3865–3868.

13 J. P. Perdew, K. Burke and M. Ernzerhof, *Phys. Rev. Lett.*, 1997, **78**, 1396–1396.

14 N. C. Handy and A. J. Cohen, *Mol. Phys.*, 2001, **99**, 403–412.

15 S. Grimme, C. Bannwarth and P. Shushkov, *J. Chem. Theory Comput.*, 2017, **13**, 1989–2009.

16 AMS DFTB 2019.3, SCM, Theoretical Chemistry, Vrije Universiteit, Amsterdam, The Netherlands, http://www.scm.com, .

17 C. Bannwarth, E. Caldeweyher, S. Ehlert, A. Hansen, P. Pracht, J. Seibert, S. Spicher and S. Grimme, *WIREs Computational Molecular Science*, **n/a**, e01493.

18 G. Santra, N. Sylvetsky and J. M. L. Martin, *J. Phys. Chem. A*, 2019, **123**, 5129–5143.

19 A. Förster and L. Visscher, *J. Comput. Chem.*, 2020, **41**, 1660–1684.
